# Supplementary material for: MED15 prion-like domain forms a coiled-coil responsible for its amyloid conversion and propagation
Source: Commun Biol. 2021 Mar 26;4:414. doi: 10.1038/s42003-021-01930-8 (PMC7997880; doi:10.1038/s42003-021-01930-8)
Supplement: Supplementary file 2 — Supplementary Information [file 42003_2021_1930_MOESM2_ESM.pdf]

SUPPLEMENTARY MATERIAL

MED15 prion-like domain forms a coiled-coil responsible for its amyloid conversion and propagation

Cristina Batlle[1], Isabel Calvo[2], Valentin Iglesias[1], Cian Lynch[2], Marcos Gil-Garcia[1], Manuel Serrano[2,3] and Salvador Ventura[1]*

[1]Institut de Biotecnologia i Biomedicina and Departament de Bioquímica i Biologia Molecular, Universitat Autónoma de Barcelona, Bellaterra, 08193, Spain

[2]Institute for Research in Biomedicine (IRB Barcelona), The Barcelona Institute of Science and Technology, Barcelona, Spain

[3]Catalan Institution for Research and Advanced Studies (ICREA), Barcelona, Spain

*Correspondence: salvador.ventura@uab.es

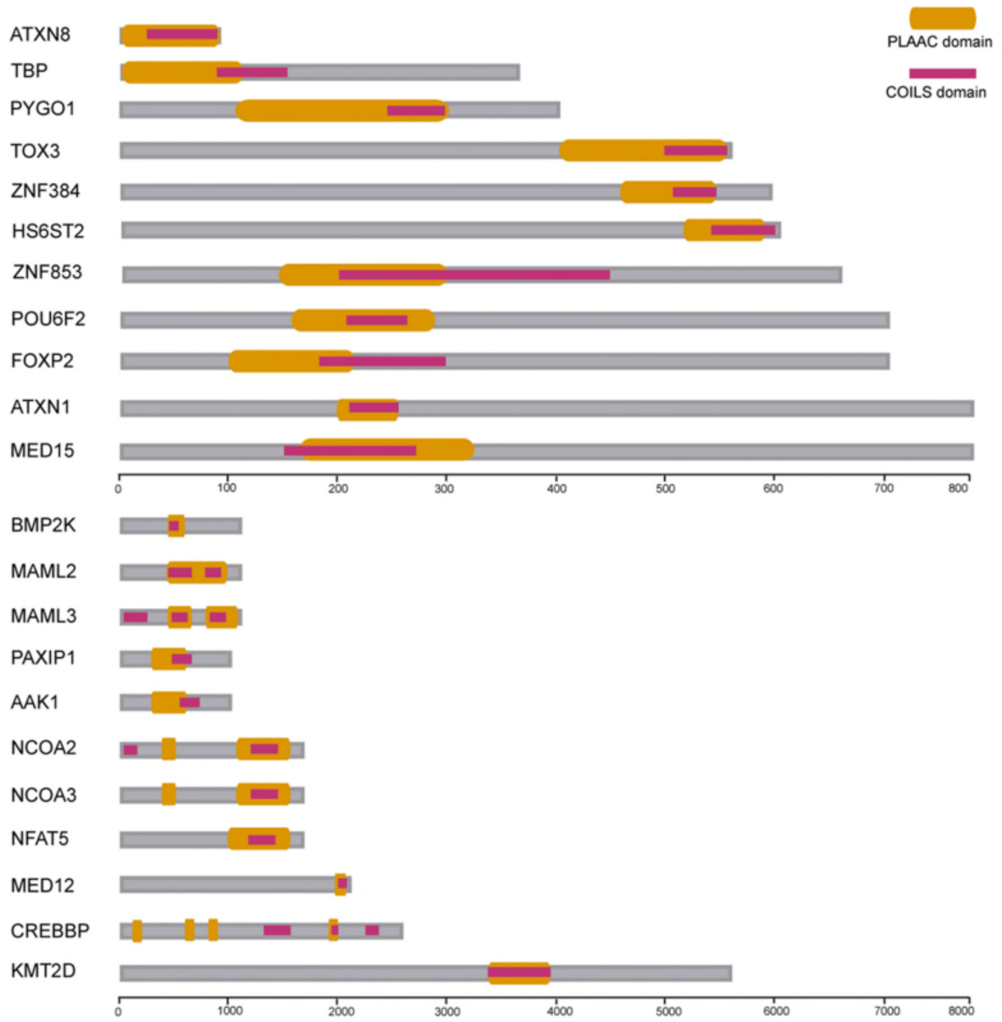

Figure S1. Human proteins with PrLDs and CC regions

List of human protein candidates with PrLDs predicted with PLAAC[1] (orange) and CC regions predicted with COILS[2] (pink).

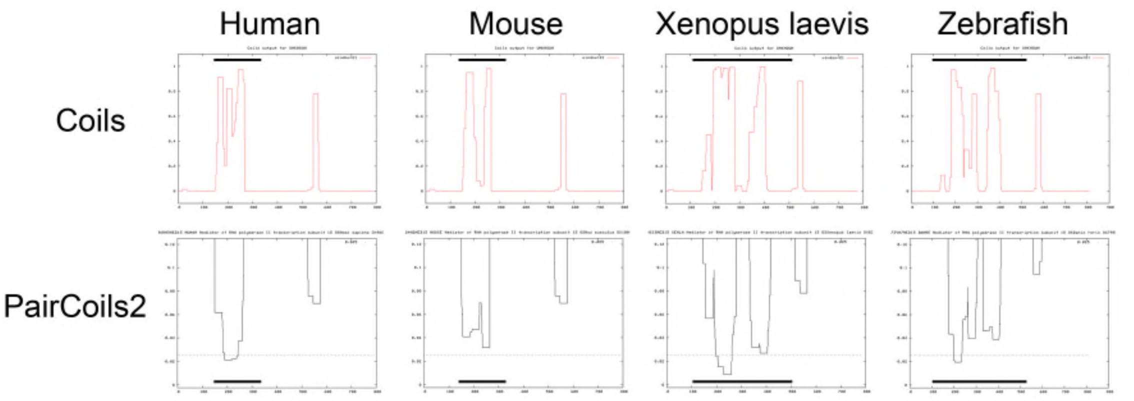

Figure S2. MED15 orthologs coiled-coil prediction.

Coils[2] and PairCoils2[3] predictions (21-residue window) of MED15 orthologs: human, mouse, xenopus laevis and zebrafish. Black rectangle indicates approximate PrLD position predicted by PLAAC[1].

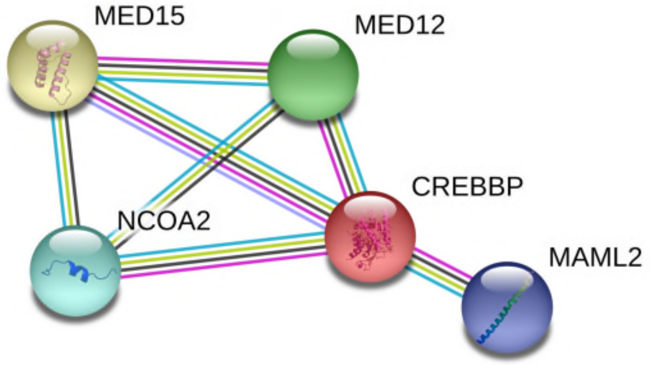

Figure S3. MED15 interaction network.

STRING[4] prediction of MED15, MED12, NCOA2, CREBBP and MAML2 interactions.

```
CLUSTAL O(1.2.4) multiple sequence alignment

SP|Q96RN5|MED15_HUMAN MDVSGQETDWRSTAFRQKLVSQIEDAMRKAGVAHSKSSKDMESHVFLKAKTRDEYLSLVA 60
SP|Q924H2|MED15_MOUSE MDVSGQETDWRSAAFRQKLVSQIEDAMRKAGVAHSKSSKDMESHVFLKAKTRDEYLSLVA 60
SP|Q6NS15|MED15_XENLA ---MDADSDWRSAPFRQKLVSQIDEAMRKAGVAHTKSSKDMESHVFLKAKTREEYLSLVA 57
SP|Q7ZVN7|MED15_DANRE MDVPGPDSDWRSPAFRQKVVAQIEEAMRKAGTGHTKSSTEMESHVFTKAKTREEYLSMVA 60
                          . ::****  ****:*:**::******..*:***.:****** *****:****:**

SP|Q96RN5|MED15_HUMAN RLIIHFRDIHNKKSQASVSDPMNALQSLTGGPAAGAAGIGMPPRGPGQSLGGMGSLGAMG 120
SP|Q924H2|MED15_MOUSE RLIIHFRDIHNKKSQASVSDPMNALQSLTGGPTPGAAGIGMPPRGPGQSLGGMGGLGAMG 120
SP|Q6NS15|MED15_XENLA RLIIHFRDILNKKSQAAATDPMNALQNLTGTAVVGGQGMGMGVRPQGAQMGAMSGMAQMA 117
SP|Q7ZVN7|MED15_DANRE RLIIHFRDIHKKAQ--GGPDPINALQNLPGGVPG-----VIGPRPPGAQMGGMGQMSMGP 113
                      ********* :* .  .  **:****.* *          :  *  * .:*.*. :.

SP|Q96RN5|MED15_HUMAN QPMSL--SGQPPPGTSGMAPHSMAVVSTATPQTQLQLQQVALQQQQQQQ---FQQ---Q 172
SP|Q924H2|MED15_MOUSE QPLPL--SGQPPPGTSGMAPHGMAVVSTATPQTQLQLQQVALQQQQQQQQQQQFQQ---Q 175
SP|Q6NS15|MED15_XENLA QQMNL--PGQPQPGASGMAPHGITGVAAGNQATQLQLQQIAQHQQQQFQQQQQQAALQHQ 175
SP|Q7ZVN7|MED15_DANRE HAMQGVAGGQQGAGAAG-------------PMQQMIQQQQQQQQQQQQQQSIQFQTFQPQ 160
                      : :     **   *::*                *:  **   :**** **         *

SP|Q96RN5|MED15_HUMAN --QQAALQ---------------------QQQ----------QQQQQQQFQAQQSAMQQQ 199
SP|Q924H2|MED15_MOUSE --Q-AALQQQ-----------------QQQQQ----------QQQQQQQFQAQQNAMQQQ 205
SP|Q6NS15|MED15_XENLA QQQFQVAQQ----QQFQVVAVAAAQQQQQQQQQ---------QQQQQQQVQ----QQQQQ 218
SP|Q7ZVN7|MED15_DANRE QQQQATMQSQQPSAMFQQIRLQQLQQHHQNQQMQHQNQQQQQAQNQQQQNQLHQTRMQQQ 220
                        *  . *                     :**           *:**** *      ***

SP|Q96RN5|MED15_HUMAN FQAVVQQQQQLQQQQQQQQHLIKLHHQNQ-----QQIQQQQQQLQRIAQLQLQQQQQQQQ 254
SP|Q924H2|MED15_MOUSE FQAVVQQQQL--QQQQQQQHLIKLHHQSQ-----QQQ-IQQQQLQRMAQLQLQQQQ---- 253
SP|Q6NS15|MED15_XENLA LQAAAQ-----------QQHILKLQLQQQQNQQQQLQQQQQQQLQRIAHLQQMQQMQQQQ 267
SP|Q7ZVN7|MED15_DANRE LQL-----QQLQQQQQQQLHQQQVHAQAQ-----AQAQAQA-QAQAQAQAQAQAQAQAQA 269
                      :*               * *  ::: * *          *  * *  *: *   *

SP|Q96RN5|MED15_HUMAN QQQQQQQQALQAQPPIQQPPMQQPQPPPSQALPQQLQQMHHTQHHQPPPQPQQPPVAQNQ 314
SP|Q924H2|MED15_MOUSE --QQQQQQALQAQPPMQQPSMQQPQPPPSQALPQQLSQLHHPQHHQPPPQAQQSPIAQNQ 311
SP|Q6NS15|MED15_XENLA QQQQQQQQQQQQQP------------PPQQV--MQLQQMQQQQVAQSQQ--QQLLSTQPQ 311
SP|Q7ZVN7|MED15_DANRE --------QAQAQAQAQ-------AQAQAQAQAQSIQQMQQQQQLQVQAQGQPQVQGQGG 314
                                * *                *.   .:.*::: *  *     *     *

SP|Q96RN5|MED15_HUMAN PSQLPPQSQTQPLVSQAQALPGQ---M-LY-TQ-PPLKFVRA---PMVVQQPPVQPQVQ- 364
SP|Q924H2|MED15_MOUSE PPQIPPQSQSQPLVSQAQALPGP---M-LY-AAQQQLKFVRA---PMVVQQPQVQPQVQ- 362
SP|Q6NS15|MED15_XENLA A----------PSLVAQGQIPSQVMPVTLTPQQQQQLKILQQVRAQQQQ---QAQHQAQQ 358
SP|Q7ZVN7|MED15_DANRE AVQMPPHSQQQQVLVP-QMVQGQHSQMSAL-SQQQQLKLQQAMQARMQQQQQQQQQQQQQ 372
                                   :     : .    :         **: :             * * *

SP|Q96RN5|MED15_HUMAN ----------------QQQ------TAVQTAQAAQMVAPGVQMITEALAQGGMHIRARFP 402
SP|Q924H2|MED15_MOUSE ----------------QVQPQVQPQAAVQAAQSAQMVAPGVQMIAEALAQGGMHVRARFP 406
SP|Q6NS15|MED15_XENLA QAQQQAHQQAQQQAQQQAQQQAAQQQAQQAAQQAQQQA-----------QQ--------- 398
SP|Q7ZVN7|MED15_DANRE QQQQQQQQQQQQQQQQQQQQQHQQQQVQQVQQASQL----TAVPGQMMPRPGMQIPPRLP 428
                                      * *       . *. * :*              :

SP|Q96RN5|MED15_HUMAN PTTAVSAIPSSSIPLGRQPMAQVSQSSLPMLSSPSPGQQVQTPQSMPPPPQPSPQPGQPS 462
SP|Q924H2|MED15_MOUSE PTSTMSAGPSSSISLGGQPTTQVSQSSLTMLSSPSPGQQVQTPQSMPPPPQPSPQPG--- 463
SP|Q6NS15|MED15_XENLA -------AAQQQAAQAHLAAGQVTQNSIPVMSSPSPVQQVQTPQPMPPPPQPSPQPSQPM 451
SP|Q7ZVN7|MED15_DANRE RATPNSAIPQNPVAIGGQQMPQAQ----QMMSSPSP-VQVQTPQSMPPPPQPQPSPQPPS 483
                               ..    .     *.      ::*****  ****** *******.*.*

SP|Q96RN5|MED15_HUMAN SQPNSNVSSGPAPSPSSFLPSPSPQPSQSPVTARTPQNF--SVPSPGPLNTPVNPSSVMS 520
SP|Q924H2|MED15_MOUSE SQPNSNVSSGPAPSPSSFLPSPSPQPSQSPVTARTPQNF--SVPSPGPLNTPVNPSSVMS 521
SP|Q6NS15|MED15_XENLA SQPNSNVSSGPAPSPSSFMPSPSPQPSQSPASARTPQNF--SVPSPGPLNTPGNPNSVMS 509
SP|Q7ZVN7|MED15_DANRE SQPNS-VSSGPTPSPGGFQPSPSPQPSQSPASSRTPQSYPLQVPSPGPLNTPGNPSSVMS 542
                      ***** *****:***..* ***********.::****.:  .********** **.****

SP|Q96RN5|MED15_HUMAN PAGSSQAEEQQYLDKLKQLSKYIEPLRRMINKIDKNEDRKKDLSKMKSLLDILTDPSKRC 580
SP|Q924H2|MED15_MOUSE PAGSSQAEEQQYLDKLKQLSKYIEPLRRMINKIDKNEDRKKDLSKMKSLLDILTDPSKRC 581
SP|Q6NS15|MED15_XENLA PASNNQSEEQQYLDKLKQLSKYIEPLRRMINKIDKNEERKKDLSKMKSLLDILTDPNKRC 569
SP|Q7ZVN7|MED15_DANRE PAGASQSEDQLYMDKLRQLSKYIEPLRRMINKIDKNEDRKKDLSKMKSLLNILTDPNTRC 602
                      **. .*:*:* *:***:********************:************:*****..**

SP|Q96RN5|MED15_HUMAN PLKTLQKCEIALEKLKNDMAVPTPPPPPVPPTKQQYLCQPLLDAVLANIRSPVFNHSLYR 640
SP|Q924H2|MED15_MOUSE PLKTLQKCEIALEKLKNDMAVPTPPPPPVLPTKQQDLCQPLLDAVLANIRSPVFNHSLYR 641
SP|Q6NS15|MED15_XENLA PLKTLQKCEIALEKLKNDMAVPTPPPPTVPSTKQQYLCQPLLDAVLANIRSPVFNHSLYR 629
SP|Q7ZVN7|MED15_DANRE PLKTLQKCEIALEKLKNDMAVPTPPPPPVPCTKKQYLCQPILDAVLANIRSPVFNHSLYR 662
                      *************************** *  **:* ****:*******************

SP|Q96RN5|MED15_HUMAN TFVPAMTAIHGPPITAPVVCTRKRRLEDDERQSIPSVLQGEVARLDPKFLVNLDPSHCSN 700
SP|Q924H2|MED15_MOUSE TFVPAMMAIHGPPIVSPVVCSRKRRFEEDERQSIPNVLQGEVARLDPKFLVNLDPSHCSN 701
SP|Q6NS15|MED15_XENLA TFMPAMTAIHGQPIASQLVVPRKRKFEEDERQSIPNVLQGEVAKLHSKFLVNLDPSHCSN 689
SP|Q7ZVN7|MED15_DANRE TFAPAMTAIHGPQITGPSIPSRKRKLEDDERQAIPNILQGEVARLNSKFLVNLDPSFCSN 722
                      ** *** ****  *..  :  ***::*:****:**.:******:*. *********.***

SP|Q96RN5|MED15_HUMAN NGTVHLICKLDDKDLPSVPPLELSVPADYPAQSPLWIDRQWQYDANPFLQSVHRCMTSRL 760
SP|Q924H2|MED15_MOUSE NGTVHLICKLDDKDLPSVPPLELSVPADYPAQSPMWIDRQWQYDANPFLQSVHRCMTSRL 761
SP|Q6NS15|MED15_XENLA NGTVYLICKLDDKNLPSVPPLQLSVPADYPDQSPLWMDNPRDYEANPFLQSVYRYMTSKL 749
SP|Q7ZVN7|MED15_DANRE NGMVHLICKLDDKNLPSVPPLQLSIPADYPDQSPHWEDDGQQYEANPFLRTVHKNMTSKL 782
                      ** *:********:*******:**:***** *** * *   :*:*****::*:: ***:*

SP|Q96RN5|MED15_HUMAN LQLPDKHSVTALLNTWAQSVHQACLSAA 788
SP|Q924H2|MED15_MOUSE LQLPDKHSVTALLNTWAQSIHQACLSAA 789
SP|Q6NS15|MED15_XENLA LQLPDKHSLTALLNTWAQSIRQACLSAA 777
SP|Q7ZVN7|MED15_DANRE LQLPDKHSVTALLNTWAQSVRQACLSA- 809
                      ********:**********::******
```

Figure S4. MED15 orthologs clustal alignment.

Clustal alignment[5] of human, mouse, xenopus laevis and zebrafish MED15 sequences. The Pro residues flanking the polyQ tracts are in red. The KIX domain is in bold and highlighted in grey.

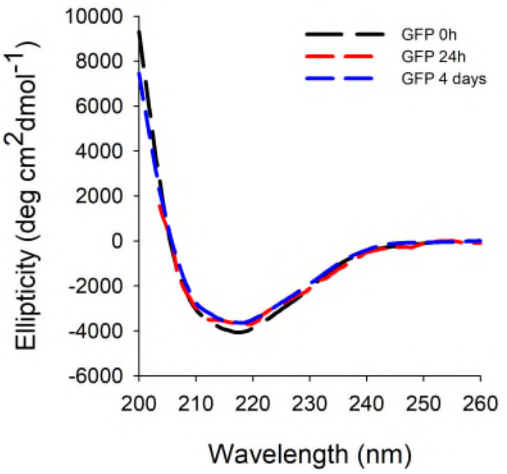

Figure S5. GFP keeps its secondary structure after 4 days

Circular dichroism of soluble (t=0h, black) and incubate (t=24h, red, or t=4 days, blue) 5 μM GFP.

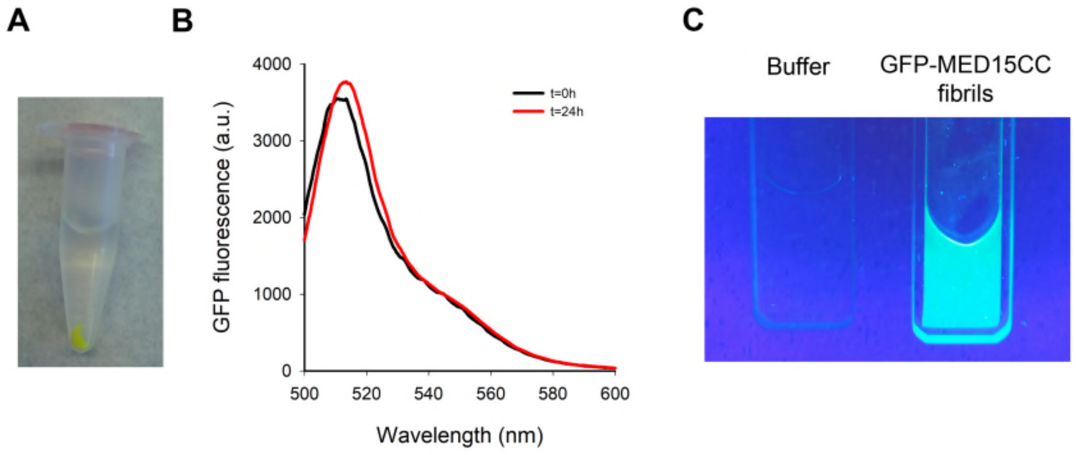

Figure S6. GFP-MED15CC keeps the native GFP fold within the fibril phase.

A) Visualization of aggregated GFP-MED15CC pellet after centrifugation. **B)** GFP fluorescence of 5 μM GFP-MED15CC at 0 and 24 h after incubation at 37ºC in 20 mM Tris pH 7.5 and 150 mM NaCl. GFP-MED15CC fluorescence at t=24 h corresponds to the pellet fraction after centrifugation and resuspended in fresh buffer. **C)** Image under UV of aggregated GFP-MED15CC pellet after centrifugation and resuspended in fresh buffer. Buffer alone is shown as control.

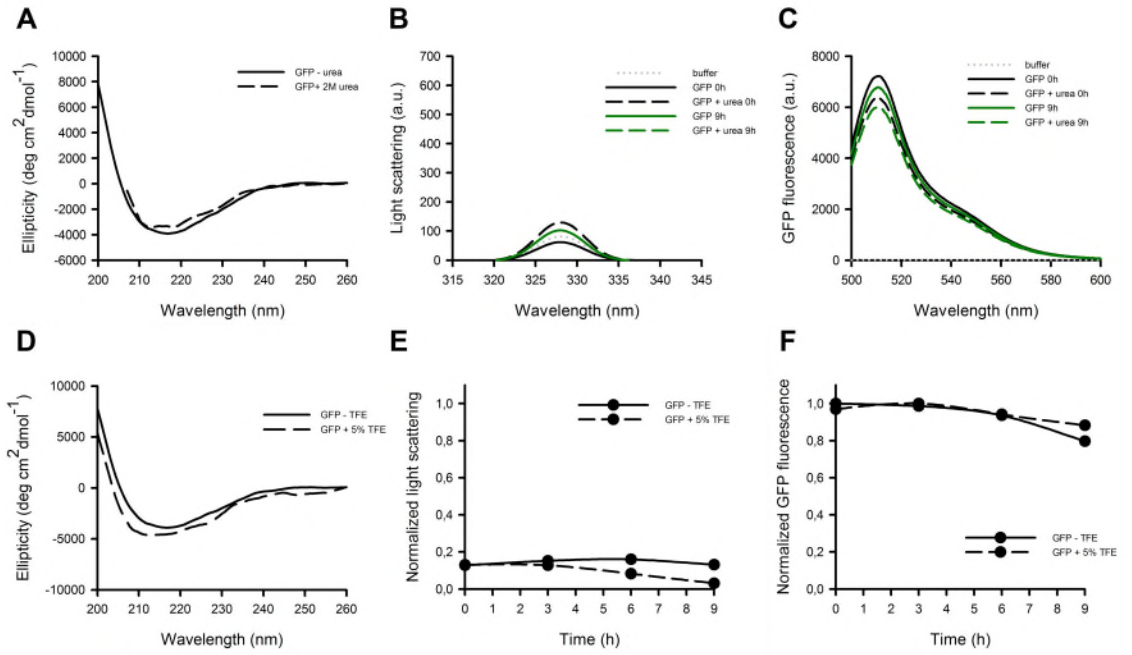

Figure S7. GFP properties in the presence of urea or TFE.

A) Far-UV CD of 5 µM GFP in the presence or absence of 2 M urea. **B)** Synchronous light scattering and **C)** GFP fluorescence of 5 µM GFP at 0 and 9 h after incubation at 37ºC in 20 mM Tris pH 7.5 and 150 mM NaCl incubated in the presence or absence of 2 M urea. **D)** Far-UV CD of 5 µM GFP in the presence or absence of 5% TFE. **E)** Synchronous light scattering and **F)** GFP fluorescence aggregation kinetics of 5 µM GFP incubated in 20 mM Tris pH 7.5 and 150 mM NaCl in the presence or absence of 5% TFE at the indicated time points.

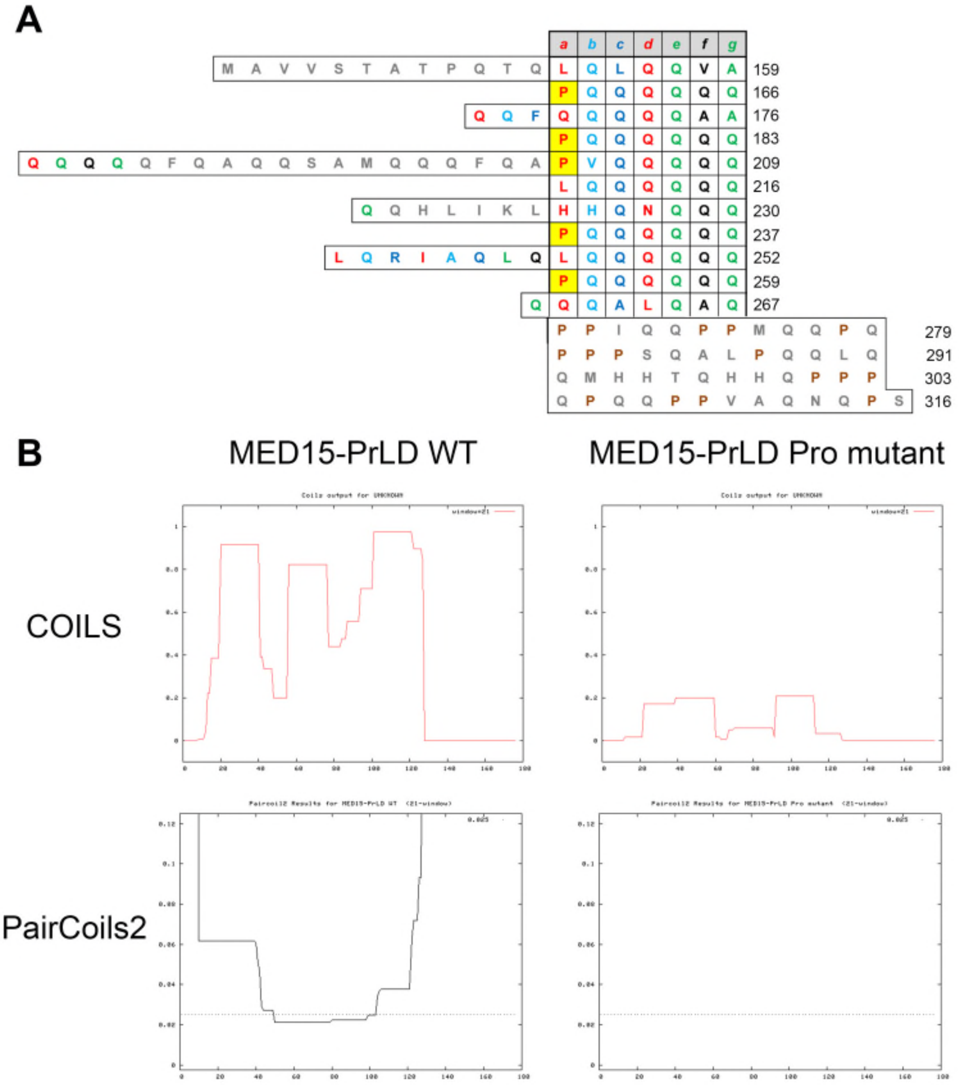

Figure S8. MED15 Pro mutations affect MED15-PrLD coiled-coil propensity.

A) Schematic diagram of the heptad repeats (a-b-c-d-e-f-g) predicted by COILS [2] (14-residue window) of the MED15-PrLD. Residues mutated to Pro in GFP-MED15PP mutant are shown and highlighted in yellow. **B)** Coils and PairCoils2 predictions of MED15 PrLD WT and Pro mutant.

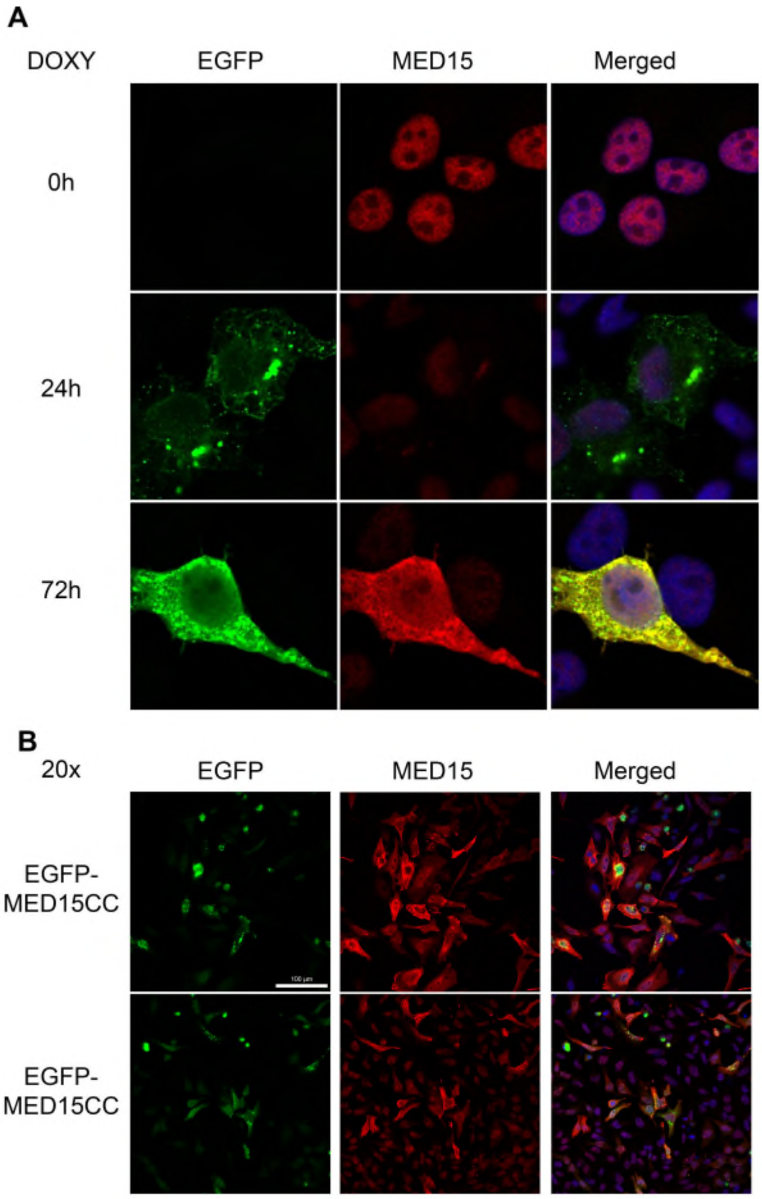

Figure S9. MED15-PrLD forms insoluble cytoplasmic inclusions in mammalian cells.

A) Cellular localization by immunofluorescence of EGFP-MED15CC in HeLa cells after addition of doxycycline (doxy) for 0, 24 or 72 h. **B)** Cellular localization by immunofluorescence of EGFP-MED15CC in HeLa cells after transient expression for 24 h. In both A and B, cells were stained with MED15 antibody (red) to evaluate the effect of transfection in the endogenous mediator subunit, and with DAPI (blue) as nuclear marker.

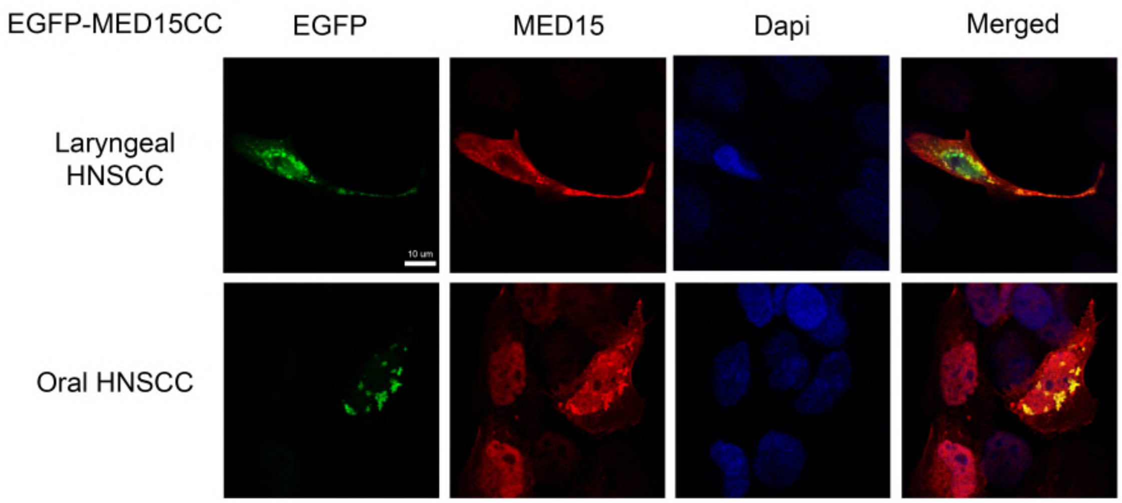

Figure S10. MED15 localization in laryngeal and oral squamous cell carcinoma.

Cellular localization by immunofluorescence of EGFP-MED15CC in laryngeal and oral squamous cell carcinoma (HNSCC) after expression for 24 h. Cells were stained with MED15 antibody (red) to evaluate the effect of transfection in the endogenous mediator subunit, and with DAPI (blue) as nuclear marker.

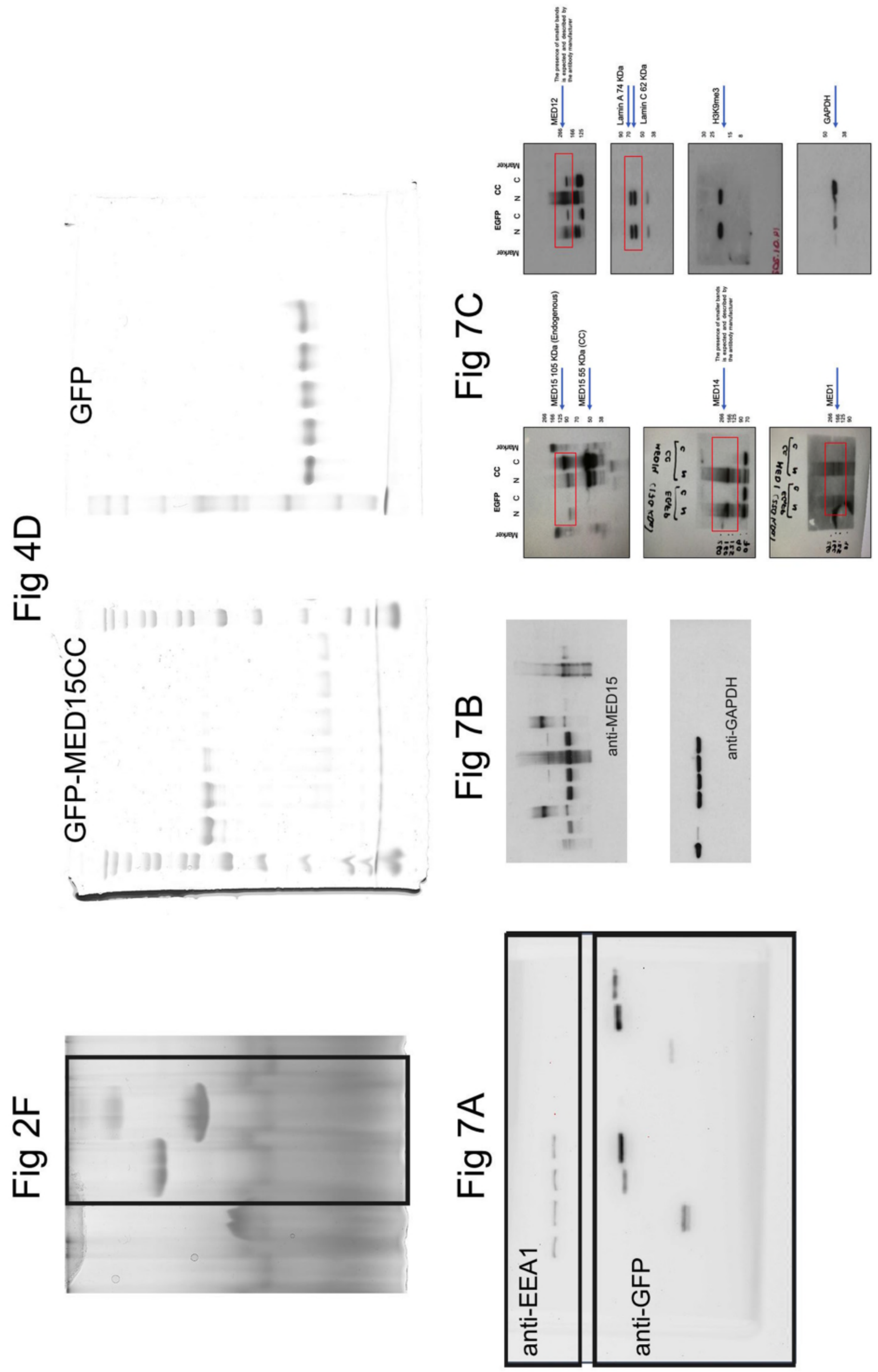

Figure S11. Raw SDS-PAGE and Western Blot data
Raw SDS-PAGE gels and Western Blots shown in the main figures.

References of Supplementary Material:

1. Lancaster, A. K., Nutter-Upham, A., Lindquist, S. & King, O. D. PLAAC: A web and command-line application to identify proteins with prion-like amino acid composition. *Bioinformatics* **30**, 2501–2502 (2014).
2. Lupas Andrei, Van Dyke Marc & Stock Jeff. Predicting coiled coils from protein sequences. *Science (80-.).* **252**, 1162–1164 (1991).
3. McDonnell, A. V, Jiang, T., Keating, A. E. & Berger, B. Paircoil2: improved prediction of coiled coils from sequence. *Bioinformatics* **22**, 356–8 (2006).
4. Szklarczyk, D. *et al.* The STRING database in 2017: Quality-controlled protein-protein association networks, made broadly accessible. *Nucleic Acids Res.* **45**, D362–D368 (2017).
5. Madeira, F. *et al.* The EMBL-EBI search and sequence analysis tools APIs in 2019. *Nucleic Acids Res.* **47**, W636–W641 (2019).
