# Supplementary material for: MED15 prion-like domain forms a coiled-coil responsible for its amyloid conversion and propagation
Source: Commun Biol. 2021 Mar 26;4:414. doi: 10.1038/s42003-021-01930-8 (PMC7997880; doi:10.1038/s42003-021-01930-8)
Supplement: Supplementary file 3 — Description of Additional Supplementary Files [file 42003_2021_1930_MOESM3_ESM.pdf]

Description of Additional Supplementary Files

Supplementary Data 1
Sheet 1) PLAAC positive reviewed human gene candidates predicted to have PrLD. **Sheet 2)** Human PrLD predicted in sheet 1 with Coils prediction score above 0.2 indicating the presence of CC region. **Sheet 3)** Function of the candidates with PrLDs and CC regions. **Sheet 4)** GO terms of the candidates with PrLDs and CC regions.

Supplementary Data 2
Sheet 1) Mouse MED15 interactors homology with its human homologue. List of interactors extracted from [1]. **Sheet 2)** List of MED15 interactors with CC regions predicted with Coils at score above 0.2. Their function is also listed. **Sheet 3)** GO terms of MED15 interactors with CC regions.

Supplementary Data 3
Raw data of all experiments performed in this publication. Each figure raw data is shown in separate sheets.

1. Quevedo, M. et al. Mediator complex interaction partners organize the transcriptional network that defines neural stem cells. Nat. Commun. 10, 2669 (2019).
